# Characterization of antimicrobial resistance and genome of a *Salmonella enterica* serovar Rissen (FARPER-637) isolated from swine in Peru

**DOI:** 10.1128/mra.01314-25

**Published:** 2026-01-14

**Authors:** Angela Montalván-Avalos, Luis Tataje-Lavanda, Doris Villanueva-Pérez, Dora Rios-Matos, Diego Paredes-Inofuente, Suly Montoya-Ortiz, Manuel Albetis-Apolaya, Ronnie Gavilan-Chavez, Manolo Fernández-Sánchez, Manolo Fernández-Díaz

**Affiliations:** 1 Laboratorios de Investigación y Desarrollo, FARVET SAC 712594 , Chincha Alta, Ica, Perú; 2 Escuela Profesional de Tecnología Médica, Universidad Privada San Juan Bautista, Lima, Perú; 3 Escuela Profesional de Medicina Humana, Universidad Privada San Juan Bautista, Lima, Perú; 4 Instituto Nacional de Salud 54719, Lima, Perú; University of Manitoba, Winnipeg, Manitoba, Canada

**Keywords:** *Salmonella*, antimicrobial resistance, molecular characterization, mechanisms of resistance, serovar Rissen

## Abstract

We report the antimicrobial susceptibility profile and genomic features of the *Salmonella enterica* serovar Rissen strain FARPER-637 isolated from Peruvian swine in 2025. Nanopore sequencing revealed blaTEM-1B, tet(A), and qnrB19. Phenotype–genotype concordance was observed except for colistin, which lacked known plasmid-mediated resistance determinants.

## ANNOUNCEMENT

*Salmonella enterica* serovar Rissen is an emerging zoonotic serovar increasingly associated with foodborne infections and antimicrobial resistance worldwide (1, 2). Although detected in porcine production systems in multiple regions, its presence and genomic characterization in Peru remain poorly documented (3, 4). Here, we describe the antimicrobial susceptibility profile and whole-genome sequence (WGS) of the strain FARPER-637, isolated within a national antimicrobial resistance surveillance initiative conducted in 2025 (5).

FARPER-637 was isolated from a rectal swab collected from clinically healthy swine in a commercial farm located in the province of Arequipa, Arequipa region, southern Peru. The samples were collected in buffered peptone water (BPW), at a ratio of 1:9, and were transported at 4°C. Conventional microbiological methods were applied for isolation, including selective enrichment in Rappaport–Vassiliadis broth from BPW; isolation in xylose lysine deoxycholate agar using 10-fold serial dilutions; biochemical identification from isolated colonies using the Analytical Profile Index (API 20E) system, obtaining biochemical profile 6,304,752, typical of the *Salmonella* genus; and PCR targeting *invA* performed from purified colonies (6–8). Antimicrobial susceptibility testing (AST) was performed using the Kirby–Bauer disk diffusion method on Mueller-Hinton agar following CLSI M100-2025, incubated at 37°C for 18–24 h in aerobic conditions (9). The isolate exhibited resistance to ampicillin, tetracycline, doxycycline, and colistin; intermediate susceptibility to cefoxitin and nalidixic acid; and susceptibility to β-lactams, aminoglycosides, carbapenems, amphenicols, trimethoprim–sulfamethoxazole, fosfomycin, and ciprofloxacin.

Genomic DNA was extracted with the DNeasy Blood and Tissue Kit (Qiagen, Germany) according to the manufacturer’s protocol. DNA integrity was evaluated by 0.8% agarose gel electrophoresis and quantified with a Qubit 4.0 fluorometer (Invitrogen, USA). WGS was performed on 200 ng of DNA. The library was prepared with the Rapid Barcoding Kit 24 (SQK-RBK114.24, Oxford Nanopore Technologies, ONT) and sequenced on R10.4.1 flow cell using MinION Mk1B device (ONT) under super-accuracy basecalling mode. Sequencing produced 90,575 raw reads, of which 78,129 passed quality filters.

Unless otherwise specified, all software was run with default parameters. All analyses from raw read processing through *de novo* assembly were performed via the GalaxyTrakr (10) web interface. Reads were processed using Porechop (v0.2.4) (11) for adapter trimming, NanoFilt (v0.1.0) (12) (-q 10 –length 500), and FastQC (13) with MultiQC (14) for quality inspection. *De novo* assembly was generated with Flye (v2.9.6) (15) (--nano-corr, -i 3, --meta), yielding five contigs, including a closed ~4.9 Mb chromosome. Assembly metrics were five contigs, N50 = 4,913,518 bp, and GC content 52%. Coverage was chromosome, 111× (circular); plasmid 1, 41,827 bp (457×, circular, IncX1); plasmid 2, 698 bp (3,854×, linear); plasmid 3, 4,819 bp (867×, circular); plasmid 4, 680 bp (3,858×, linear).

Final consensus polishing was performed locally with Medaka (v2.1.1) (16) (-m dna_r10.4.1_e8.2_400bps_sup@v5.2.0), following the standard workflow medaka_consensus. Annotation employed NCBI PGAP (17). Serovar prediction by SISTR (v1.1.1) (18) confirmed *S. enterica* serovar Rissen, and SeqSero2 identified antigenic profile 7:f,g:–. Antimicrobial resistance genes were detected with staramr (v0.10.0) using the following database versions ResFinder (072621; DB date 24 May 2022), PointFinder (072621.2; DB date 1 Feb 2021), PlasmidFinder (DB date 18 Jan 2023), and MLST 2.23.0. Detected AMR determinants included *blaTEM-1B*, *tet(A*), and *qnrB19*, all located on an IncX1 plasmid. No *mcr* genes were identified.

Genotype–phenotype concordance was consistent: *blaTEM-1B* correlated with resistance to ampicillin, *tet(A*) with tetracycline and doxycycline resistance, and *qnrB19* with borderline quinolone effects. These findings agreed with the observed phenotypic resistance to β-lactams, tetracycline, doxycycline, and quinolone borderline effects. Colistin resistance lacked known plasmid-mediated *mcr* genes, suggesting the involvement of chromosomal or alternative resistance mechanisms. Table 1 summarizes phenotype–genotype concordance.

**TABLE 1:** Phenotypic antimicrobial susceptibility and corresponding genomic determinants in *S. enterica* serovar Rissen FARPER-637

Antimicrobial/class	AST result	Genomic determinant(s) detected	Interpretation
Ampicillin	Resistant	*blaTEM-1B (IncX1 plasmid*)	Genotype matches phenotype
Tetracycline	Resistant	*tet(A*)	Genotype matches phenotype
Doxycycline	Resistant	*tet(A*)	Genotype matches phenotype
Ciprofloxacin	Sensitive (but borderline via *qnrB19*)	*qnrB19; parC T57S*	Chromosomal/plasmid determinants present, not fully expressed phenotypically
Nalidixic acid	Intermediate	*qnrB19; parC T57S*	Genotype consistent with reduced quinolone susceptibility
Colistin	Resistant	None (no *mcr* genes detected)	Phenotype unexplained by known plasmid-mediated colistin genes
β-lactams (AMC, CXM, CTX, CRO, and CAZ)	Sensitive	*blaTEM-1B* present but no *ESBL* genes	Genotype consistent (no ESBL; AMC protects)
Carbapenems (IPM and MEM)	Sensitive	None detected	Fully concordant
Aminoglycosides (GM and MK)	Sensitive	None detected	Fully concordant
Chloramphenicol/florfenicol	Sensitive	None detected	Fully concordant
TMP-SMX	Sensitive	None detected	Fully concordant
Fosfomycin	Sensitive	None detected	Fully concordant

This report documents the first genomic characterization of *S*. Rissen from Peruvian swine, emphasizing the need to expand genomic AMR surveillance in Latin American livestock systems (1, 19).

## Data Availability

The whole-genome sequence of *S. enterica* serovar Rissen strain FARPER-637 is available in GenBank under accession JBSBGN000000000. Raw nanopore sequencing data have been deposited in the Sequence Read Archive under accession SRR35981468. The sample is associated with BioSample SAMN53074355 and BioProject PRJNA528985.
