# Erratum for Montalván-Avalos et al., “Characterization of antimicrobial resistance and genome of a *Salmonella enterica* serovar Rissen (FARPER-637) isolated from swine in Peru”

**DOI:** 10.1128/mra.00302-26

**Published:** 2026-04-16

**Authors:** Angela Montalván-Avalos, Luis Tataje-Lavanda, Doris Villanueva-Pérez, Dora Rios-Matos, Diego Paredes-Inofuente, Suly Montoya-Ortiz, Manuel Albetis-Apolaya, Ronnie Gavilan-Chavez, Manolo Fernández-Sánchez, Manolo Fernández-Díaz

**Affiliations:** 1 Escuela Profesional de Tecnología Médica, Universidad Privada San Juan Bautista 33222, Lima, Perú; 2 Escuela Profesional de Medicina Humana, Universidad Privada San Juan Bautista 33222, Lima, Perú; 3 Laboratorios de Investigación y Desarrollo, FARVET SAC 712594 , Chincha Alta, Ica, Perú

## ERRATUM

Volume 15, no. 2, e01314-25, 2026, https://doi.org/10.1128/mra.01314-25. The affiliations and the affiliation numbers for each author should appear as shown in this erratum.

